# Anti-Inflammatory and Mineralization Effects of Bromelain on Lipopolysaccharide-Induced Inflammation of Human Dental Pulp Cells

**DOI:** 10.3390/medicina57060591

**Published:** 2021-06-08

**Authors:** Joo-Hyun Hong, Mi-Ra Kim, Bin-Na Lee, Won-Mann Oh, Kyung-San Min, Yeong-Gwan Im, Yun-Chan Hwang

**Affiliations:** 1 Department of Conservative Dentistry, Dental Science Research Institute, School of Dentistry, Chonnam National University, Gwangju 61186, Korea; juile88@naver.com (J.-H.H.); jajoongvv@naver.com (M.-R.K.); bnlee13@jnu.ac.kr (B.-N.L.); wmoh@chonnam.ac.kr (W.-M.O.); 2 Department of Conservative Dentistry, School of Dentistry, Jeonbuk National University, Jeonju 54896, Korea; endomin@gmail.com; 3 Department of Oral Medicine, Dental Science Research Institute, School of Dentistry, Chonnam National University, Gwangju 61186, Korea

**Keywords:** bromelain, human dental pulp cells, lipopolysaccharide, NF-κB, MAPK

## Abstract

*Background and Objectives*: Bromelain is a mixture of protease obtained from pineapple fruits or stems. Even though the biological mechanism of action of bromelain has not been completely understood, it is well known that bromelain possesses anticancer, anti-inflammatory and immunomodulatory effects. This study investigated the anti-inflammatory effects of bromelain on lipopolysaccharide (LPS)-induced human dental pulp cells (hDPCs). *Materials and Methods*: Cell viability after bromelain treatment was measured using WST-1 assay. We exposed hDPCs to 5 µg/mL of LPS with 2.5 or 5 µg/mL of bromelain. We performed reverse-transcription polymerase chain reaction and enzyme-linked immunosorbent assay to detect interleukin-1β, interleukin-6, and interleukin-8 levels. Western blots were used to detect intercellular adhesion molecules-1 (ICAM-1) and vascular cell adhesion molecules-1 (VCAM-1) levels. Immunofluorescence staining and Western blots were used to determine bromelain’s anti-inflammatory mechanism. We also performed alkaline phosphatase and Alizarin red staining to verify mineralization nodule formation. *Results*: Bromelain at 2.5, 5, 10, or 20 µg/mL did not affect the viability of hDPCs significantly. LPS increased interleukin-1β, interleukin-6, interleukin-8, ICAM-1 and VCAM-1 expression in hDPCs. Bromelain significantly decreased interleukin-1β, interleukin-6, interleukin-8, ICAM-1, and VCAM-1 levels in hDPCs, which were stimulated by LPS. Bromelain treatment significantly reduced p65 phosphorylation in the cytoplasm and the nucleus. It also significantly decreased phosphorylation levels of extracellular signal-related kinases (ERK) and p38 mitogen-activated protein kinases (p38). Bromelain also promoted ALP activity and mineralized nodule formation. *Conclusions*: Bromelain inhibits the expression of inflammatory cytokines in LPS-stimulated hDPCs. The inhibitory effect of bromelain on inflammatory mediators is related to decreased NF-κB and the MAPK pathway. Therefore, bromelain might have the potential to be used for regenerative endodontics, including vital pulp therapy.

## 1. Introduction

Dental pulp tissue is often infected by microorganisms through the dentinal tubule and root canal [1]. Gram-negative bacteria are present in deep caries and pulpitis. Lipopolysaccharides (LPS) are components of Gram-negative-bacterial cell walls. They play an important role as a virulence factor [2]. LPS can stimulate immune cells by Toll-like receptor 4 (TLR4), a member of the Toll-like receptor protein family. When LPS binds to TLR4, the MAPK pathway or NF-κB pathway is activated, resulting in the production of proinflammatory cytokines such as tumor necrosis factor (TNF)-α, interleukin (IL)-1β, IL-6, and IL-8 [3,4].

Bromelain is a mixture of enzymes obtained from pineapple (*Ananas comosus*) stems and fruits. Bromelain contains various thiol endopeptidases, phosphatase, glucosidase, peroxidase, cellulase, and several protease inhibitors [5]. Although bromelain’s mechanism of action has not been completely identified, many studies have demonstrated that it has anti-inflammatory, antimicrobial, and anticancer effects [6]. Bromelain can activate inflammatory mediators such as IL-1β, IL-6, INF-γ, and TNF-α in mouse and human cells [7,8]. Conversely, bromelain can reduce granulocyte colony-stimulating factor (G-CSF), IL-1β, IL-6, and TNF-α secretions when immune cells have already been stimulated in an inflammatory condition [9]. It has been shown that bromelain can inhibit nuclear factor kappa-B (NF-κB) translocation in human cancer cells by suppressing IκB phosphorylation [10]. In addition, bromelain can block the activation of the mitogen-activated protein kinase (MAPK) pathway in raw 264.7 cells [11]. Bromelain has antibacterial effects against *Enterococcus faecalis* and Porphyromonas gingivalis [12]. Bromelain had anti-inflammatory effects and reduced edema after extraction of third molars [13]. Bromelain might be able to suppress inflammation in dental pulps by inhibiting NF-κB and MAPK pathways. However, no study has reported the anti-inflammatory effects of bromelain on human dental pulp cells (hDPCs). Thus, this study aimed to investigate whether bromelain could suppress the expression of inflammatory mediators and cell adhesion molecules associated with inhibition of NF-κB and MAPK pathways in hDPCs.

## 2. Materials and Methods

### 2.1. Primary Culture of hDPCs

This study was performed under the approval of the Ethical Review Board (Chonnam National University Dental Hospital, No. 2016-009) (01/03/2016). Each patient consented to the study and provided written consent. Extracted human supernumerary teeth were obtained from the Department of Pediatric Dentistry. We isolated pulp tissues from these teeth under aseptic conditions, rinsed them with Dulbecco’s phosphate-buffered saline solution (DPBS, Welgene, Daegu, Korea), and placed them in 60-mm dishes. We cultured the cells in growth media (GM) consisting of α-minimum essential medium (α-MEM, Gibco Invitrogen, Grand Island, NY, USA) containing 10% fetal bovine serum (FBS, Gibco Invitrogen) and 1% antibiotics (100 U/mL penicillin and 100 mg/mL streptomycin, Gibco Invitrogen). We stored the plates at 37 °C in a humidified atmosphere of 5% CO_2_. We used the cultured cells between the 3rd and 6th passages. 

### 2.2. Cell Treatment

We treated the cells with bromelain (Sigma Chemical, St. Louis, MO, USA) for one hour following treatment with *Escherichia coli* (*E. coli*) LPS (Sigma-Aldrich, St. Louis, MO, USA). For mineralization-related experiments, we cultured the cells in an odontogenic induction medium (OIM) containing 50 mg/mL ascorbic acid (Sigma-Aldrich), 10 mmol/L β-glycerophosphate (Santa Cruz, Dallas, TX, USA), and 100 nmol/L of dexamethasone (Sigma-Aldrich). 

### 2.3. Cell Viability Test

We cultured the cells (1 × 10^4^) onto 96-well plates for 24 h and treated the cells with different concentrations of bromelain (2.5, 5, 10, 20 and 40 μg/mL) for 24 h. Then, we performed WST-1 assay by using an EZ-Cytox-enhanced cell viability assay kit (Daeil Lab Service, Seoul, Korea). Briefly, we applied 10 µL EZ-Cytox reagent to each well and incubated for 4 h. Then, we measured absorbance using a spectrophotometer (Thermo Scientific, Waltham, MA, USA) at 450 nm wavelength.

### 2.4. Real-Time Polymerase Chain Reaction (PCR)

We seeded the cells (2 × 10^5^) into 6-well plates. After 24 h, we treated the cells with 5 μg/mL LPS in the presence of 0, 2.5, and 5 μg/mL bromelain for 24 h. Then, we extracted total RNA using Trizol reagent (Gibco Invitrogen). We synthesized cDNA using the extracted total RNA, a random primer (Promega Biotech, Piscataway, NJ, USA), and an AccessQuick™ real-time (PCR) system (Promega, Madison, WI, USA). We performed quantitative real-time PCR with a QuantiTect SYBR Green PCR Kit (Qiagen, Valencia, CA, USA) on a 72-well Rotor-Gene 6000 (Corbett Research, Sydney, Australia). The primer sequences used for PCR are shown in Table 1. We determined relative gene expression levels using the 2^−∆∆Ct^ method [14].

### 2.5. Enzyme-Linked Immunosorbent Assay (ELISA)

We seeded the cells (2 × 10^5^) into 6-well plates. After 24 h, we treated the cells with 5 μg/mL LPS in the presence of 0, 2.5, and 5 μg/mL bromelain for 24 h. Then, we determined levels of IL-1β, IL-6, and IL-8 using ELISA kits (R & D Systems, Minneapolis, MN, USA). 

### 2.6. Western Blot Analysis

To measure cell adhesion molecules stimulated by inflammatory cytokines, we seeded hDPCs (3 × 10^5^) into 60-mm plates. We cultured the cells in GM with 5 µg/mL LPS in the presence of 0, 2.5, and 5 µg/mL bromelain for 48 h. To assess the relationship between NF-κB and MAPK pathways, we seeded hDPCs (8 × 10^5^) into 100-mm culture plates. Then, we cultured the cells in GM with 5 µg/mL of LPS in the presence of 0 and 5 µg/mL bromelain for 3 h. We harvested the cell extracts in lysis buffer (Cell Signaling, Beverly, MA, USA) and centrifuged it at 13,000 rpm for 10 min. We collected supernatants and determined protein concentrations with a Lowry protein assay reagent kit (Bio-Rad Laboratories, Hercules, CA, USA). We then separated proteins by SDS-PAGE and transferred them to PVDF membranes. After blocking using 5% non-fat dried skim milk in PBST, membranes were incubated with anti-vascular cell adhesion molecule-1 (VCAM-1), anti-intercellular adhesion molecule-1 (ICAM-1) (1:2000; Santa Cruz), anti-extracellular signal-related kinases (ERK), anti-phospho-ERK, anti-C-Jun N-terminal kinase (JNK), anti-phospho-JNK, anti-p38 mitogen-activated protein kinases (p38), anti-phosho-p38, anti-p65, anti-phospho-p65, lamin B, and β-actin (1:2000; Cell signaling). We incubated the membranes with horseradish peroxidase (HRP)-conjugated anti-rabbit IgG secondary antibodies (1:10,000 Sigma-Aldrich). Chemiluminescent HRP substrate (Millipore, Billerica, MA, USA) was used to visualize protein signals using a Chemiluminescence Imaging System (Ez-Capture; Atto, Tokyo, Japan). Nuclear and cytoplasmic extractions were conducted with an NE-PER nuclear and cytoplasmic extraction reagents kit (Thermo Scientific Pierce, Rockford, IL, USA) according to the manufacture’s instruction. We stored the cytoplasmic and nuclear supernatants at −80 °C.

### 2.7. Immunofluorescence Staining

We seeded hDPCs into 24-well plates. Then, we exposed them to 5 μg/mL LPS with or without 5 μg/mL bromelain or Bay11-7082 (Sigma-Aldrich), an inhibitor of NF-κB, for 3 h. Then, we fixed the cells with 4% paraformaldehyde for 10 min, permeabilized them with 0.1% Triton X-100 for 15 min, and blocked them with 3% BSA for 1 h. We incubated the cells with a primary antibody against p65 (1:500; Cell Signaling) overnight at 4 °C and then incubated them with an antirabbit secondary antibody (1:200; Cell Signaling) for 1 h at room temperature. Nuclei were counterstained with 4′, 6-diamidino-2-phenylindole (DAPI, Sigma-Aldrich) and stored at 4 °C in the dark. Then, we observed the cells under a confocal laser scanning microscope (CLSM) (Carl Zeiss, Oberkochen, Germany).

### 2.8. Alkaline Phosphatase (ALP) Staining and Alizarin Red Staining

We seeded hDPCs (2 × 10^4^) into 24-well plates and cultured the cells in OIM with 5 µg/mL LPS in the presence of 0, 2.5, and 5 µg/mL bromelain for 7 and 14 days. After 7 and 14 days, we removed the medium and fixed it in 70% ethanol for 1 h, followed by rinsing with distilled water. We treated the fixed cells with 300 µL ALP staining reagent (1-step NBT/BCIP solution; Thermo Fisher Scientific) or 2% alizarin red S staining reagent (LIFELINE Cell Tech, Frederick, MD, USA). We took a photograph after removing the staining reagent. For quantitative analysis, we extracted the stain 10% (*w/v*) cetylpyridinium chloride in 10 mmol/L sodium phosphate (pH 7.0) for 30 min. Then, we measured absorbance measured at 540 nm. 

### 2.9. Statistical Analysis

Data were statistically analyzed using SPSS 23.0 software program (SPSS, Chicago, IL, USA). Statistical significance was determined using a one-way analysis of variance (ANOVA). A significant difference was considered at *p* < 0.05. All experiments were carried out in triplicate.

## 3. Results

### 3.1. Effects of Bromelain on hDPC Cell Viability

**Figure 1:**
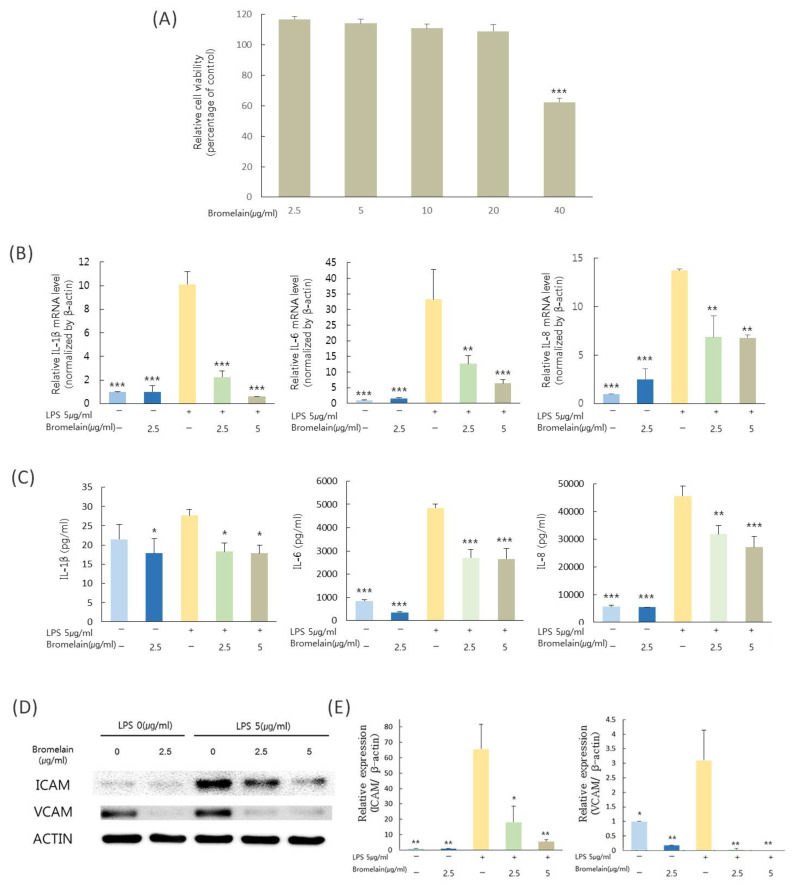
Cell viability and anti-inflammatory activity of bromelain. (**A**) Cell viability after treatment with bromelain (2.5, 5, 10, 20, 40 µg/mL) based on WST-1 assay. Cell viability in the control group (no bromelain) was used as a reference. Anti-inflammatory effects of bromelain (2.5 and 5.0 µg/mL) on LPS (5 µg/mL) induced hDPCs were determined. (**B**) Relative mRNA levels of IL-1β, IL-6, and IL-8 based. (**C**) Protein levels of IL-1β, IL-6, and IL-8 based on ELISA. (**D**,**E**) Protein levels of ICAM-1 and VCAM-1 based on Western blot analysis. * *p* < 0.05, ** *p* < 0.01, *** *p* < 0.001 compared with LPS treated group.

Figure 1A shows cell viability after treatment with bromelain (2.5, 5, 10, 20, or 40 µg/mL) based on WST-1 assay. In cell viability, there was a significant difference between the group treated with 40 µg/mL bromelain and the control (*p* < 0.05).

### 3.2. Effects of Bromelain on Levels of Proinflammatory Cytokines, mRNA Mediators, and Protein Expression in LPS-Stimulated hDPCs

To determine effects of bromelain on LPS-induced pulpal inflammation, expression and secretion of IL-1β, IL-6, and IL-8 and expression of VCAM-1 and ICAM-1 in hDPCs stimulated with *E. coli* LPS in the presence of bromelain were examined. Treatment with bromelain at 2.5 or 5 µg/mL significantly attenuated LPS-stimulated IL-1β, IL-6, and IL-8 expression in hDPCs (Figure 1B). IL-1β, IL-6, and IL-8 proteins secreted into the supernatant of LPS-stimulated hDPCs were reduced by bromelain (Figure 1C). In addition, bromelain decreased the VCAM-1 and ICAM-1 expression in hDPCs stimulated with LPS treatment (Figure 1D,E).

### 3.3. Effects of Bromelain on LPS-Stimulated hDPCs Are Dependent on NF-κB Pathway in hDPCs

**Figure 2:**
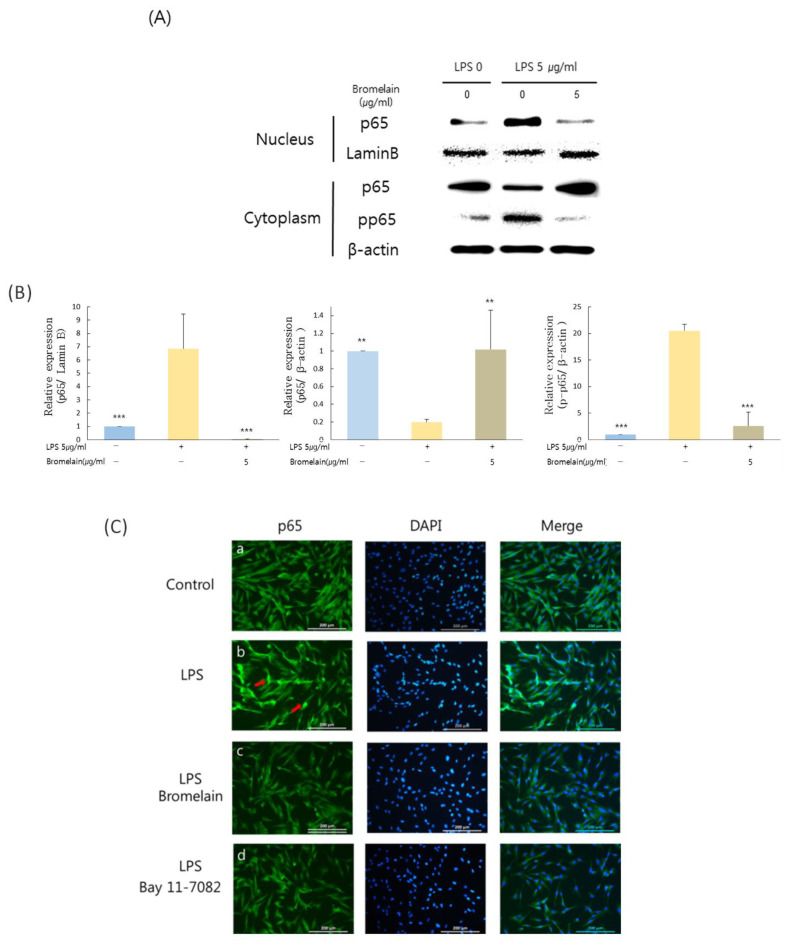
Effects of bromelain on NF-κB pathway in HDPCs. Cells pretreated with 5 µg/mL *E. coli* LPS for one hour were cultured with or without 5 µg/mL bromelain treatment for three hours. (**A**,**B**) The protein level of p65, phospho-p65 in the cytoplasm, and p65 in the nucleus of hDPCs determined by Western blot analysis. (**C**) Results of immunofluorescence staining to detect effects of bromelain on NF-κB activity. (C-a) No treatment group. (C-b) LPS treatment group. (C-c) LPS and Bromelain treatment group. (C-d) LPS and Bay 11-7082 treatment group. Green fluorescence represented that endogenous p65 was strongly expressed in the nucleus of LPS-induced hDPCs (C-b, red arrows). ** *p* < 0.01, *** *p* < 0.001 as compared to LPS treated group.

Bromelain inhibited phosphorylation levels of p65 in the cytoplasm and nuclear translocation of p65 (Figure 2A). Bromelain treatment significantly lowered the LPS-increased phosphorylation level of p65 in the cytoplasm and p65 level in the nucleus (Figure 2A,B). In immunofluorescence staining, green fluorescence suggested that endogenous p65 was strongly expressed in the nucleus of LPS-induced hDPCs (Figure 2Cb, red arrows). They were weakly observed in the nucleus following treatment with bromelain or NF-κB inhibitor, Bay11-7082 (Figure 2Cc,Cd).

### 3.4. Effects of Bromelain on LPS-Stimulated hDPCs Are Dependent on ERK and p38 Pathway in hDPCs

**Figure 3:**
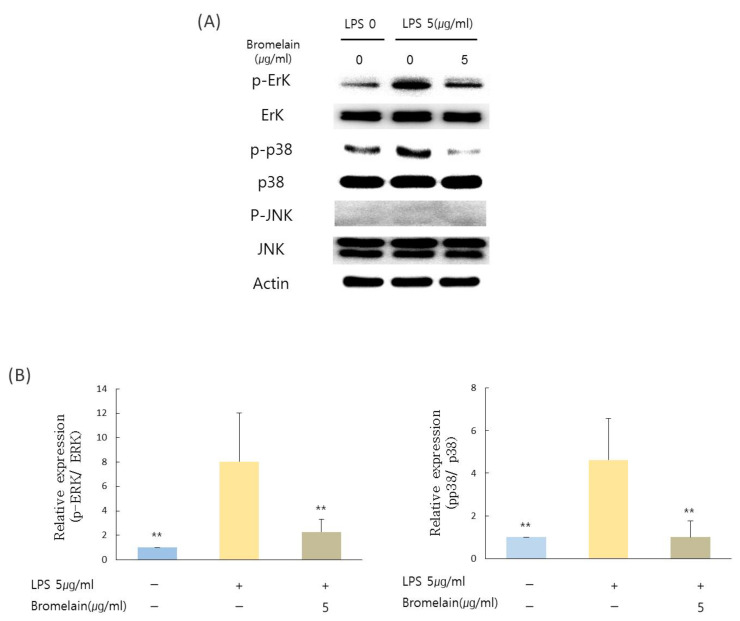
Effects of bromelain on the MAPK pathway in hDPCs. Cells pretreated with 5 µg/mL *E. coli* LPS for one hour were cultured in the presence or absence of 5 µg/mL bromelain for three hours. (**A**,**B**) Phosphorylation levels of ERK, JNK, and p38 of hDPCs were determined using Western blot analyses. Data are presented as mean values of three experiments ± S.D. ** *p* < 0.01 compared to LPS treated group.

Bromelain inhibited phosphorylation levels of ERK and p38 following three hours of treatment. Bromelain significantly decreased phosphorylation levels of ERK and p38 mitogen-activated protein kinase (p38) (Figure 3A,B). However, there was no significant change in the phosphorylation level of JNK after treatment with bromelain.

### 3.5. Effects of Bromelain on Mineralization in hDPCs

**Figure 4:**
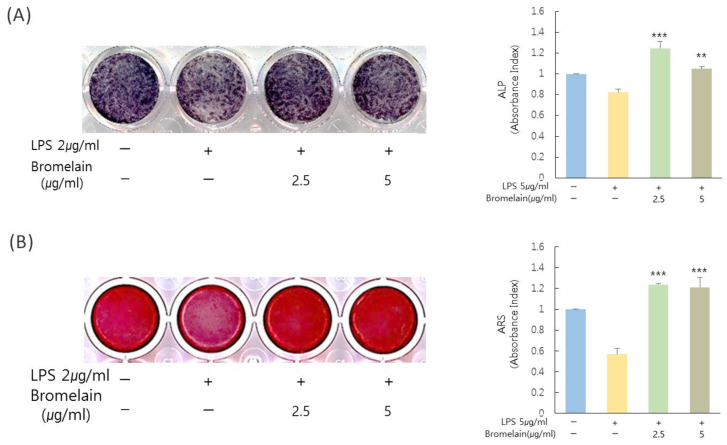
Effects of bromelain on mineralization in hDPCs. Cells were treated with *E. coli* LPS (2 µg/mL) in the presence or absence of bromelain (2.5 and 5.0 µg/mL) in OIM media. (**A**) Treatment of bromelain increased ALP staining in hDPCs than the control (LPS treatment with bromelain treatment). (**B**) Treatment with bromelain increased Alizarin red S staining in hDPCs as compared to the control. ** *p* < 0.01, *** *p* < 0.001 as compared to the control.

To verify the effect of bromelain on mineralization in hDPCs, ALP staining and Alizarin red staining were performed for cells treated with or without 2.5 and 5 µg/mL bromelain. Mineralization in LPS-stimulated hDPCs was significantly increased by treatment with bromelain (Figure 4).

## 4. Discussion

Bacterial penetration into the pulp involves inflammation, necrosis, and periapical disease [15]. LPS can mediate bacterial activity and induce an immune response. LPS is also associated with the development of pulpitis and necrosis of the pulp. It has been reported that bromelain possesses pharmacological effects in various tissues, such as tissues of patients with rheumatoid arthritis [16] and in the intestinal mucosa of the colon [9]. IL-1β and IL-6 are inflammatory cytokines that play an essential role in immune responses and the development of acute-phase inflammation [17]. IL-8 is a chemokine with a primary function to facilitate the recruitment and activation of neutrophils to sites of acute inflammation [18]. A previous study has shown that bromelain can be seen to effectively decrease neutrophil migration to sites of acute inflammation [19]. Bromelain can also inhibit IL-2, IFN- γ, and IL-4 mRNA production [20].

However, studies that evaluate the anti-inflammatory effect of bromelain in hDPCs have not been reported yet. In this study, bromelain decreased mRNA levels of IL-1β, IL-6, and IL-8 upregulated by LPS. The suppressive effect of bromelain on these inflammatory cytokines in hDPCs is similar to that of previous studies with other cell types [9,21].

ICAM-1 and VCAM-1 are members of the integrin superfamily that can mediate leukocyte adhesion and inflammatory disease [22,23]. ICAM-1 and VCAM-1 are expressed in human dental pulps that can enhance inflammation [24]. In the present study, bromelain effectively decreased protein levels of ICAM-1 and VCAM-1 upregulated by LPS.

NF-κB can regulate innate and adaptive immune reactions and mediate inflammatory responses. NF-κB can induce proinflammatory cytokine expression. Downregulation of NF-κB activation contributes to pathogenic processes of inflammatory diseases [25]. In the regulation of inflammatory processes, NF-κB pathway inhibition is very critical factor [26]. In this study, bromelain inhibited the translocation of p65 from the cytoplasm to the nucleus. Therefore, nuclear p65 increase by LPS was decreased. From these results, it appears that the anti-inflammatory effect of bromelain is inhibition of the NF-κB pathway. These results are consistent with previous results that bromelain reduced inflammatory cytokine production and apoptosis by inhibiting the NF-κB pathway in carcinoma and melanoma cells [10].

Another signaling pathway associated with inflammation is the MAPK pathway involving ERK, JNK, and p38 signaling [8]. p38 MAP kinase is a crucial mediator of LPS-induced cytokine gene expression [27]. Activation of JNK kinase and ERK 1/2 is also related to the induction of IL-1β and IL-6 in an LPS-induced inflammation model [28]. In this study, bromelain inhibited the p38-ERK pathway. However, the JNK pathway was not inhibited by bromelain in hDPCs. Conversely, bromelain can inhibit the LPS-induced JNK pathway in RAW 264.7 cells [11].

Vital pulp therapy requires biomaterials to possess specific characteristics such as biocompatibility, anti-inflammatory effects, and mineralization ability to lead to new reparative dentin. In this study, bromelain has calcium nodule formation ability in the inflammation model. Regenerative endodontics has been proposed to overcome the obstacles related to the clinical management of immature human permanent teeth with necrotic pulps [29]. Disinfection of the root canal system is a prominent success factor in regenerative endodontic treatment. Mechanical instrumentation is contraindicated because of thin dentinal walls that could increase the risk of root fracture [30]. The American Association of Endodontics (AAE) recommends irrigation using 1.5% NaOCl and EDTA and the placement of triple-antibiotic paste or calcium hydroxide paste to disinfect the root canal system and expose the dentin surface. Disinfection, anti-inflammation, and dentinal tubule exposure are important factors for regenerative endodontic treatment [31]. In this study, bromelain showed anti-inflammatory and mineralization effects. Bromelain also showed effects on deproteinization and removal of the collagen network on fresh dentin. Thus, bromelain is helpful for exposing dentinal tubules during a regenerative endodontic treatment [32]. Taken together, these results suggest that bromelain might be a potential therapeutic tool for clinical use in regenerative endodontics due to its anti-inflammatory and deproteinizing characteristics. Further in vivo and in vitro studies are needed to test the effectiveness of bromelain in dentin regeneration for its therapeutic use clinically.

## 5. Conclusions

In conclusion, Bromelain has an anti-inflammatory and mineralization effect on the LPS-stimulated inflammation in hDPCs. Therefore, Bromelain might be used as a therapeutic method for the treatment of vital pulp therapy and regenerative endodontics.

## Figures and Tables

**Table 1:** Primer sequences used for real-time PCR.

Gene	Sequences (5′-3′)
IL-1 β	Forward: TCA ATA TTA GAG TCT CAA CCC CCA
	Reverse: TTC TCT TTC GTT CCC GGT GG
IL-6	Forward: CAT CAC CAT CTT CCA GGA G
	Reverse: AGG CTG TTG TCA TAC TTC TC
IL-8	Forward: TTT CTG TTA AAT CTG GCA ACC CTA GT
	Reverse: ATA AAG GAG AAA CCA AGG CAC AGT
β-actin	Forward: CTC CTT AAT GTC ACG CAC GAT
	Reverse: CCT TGT AGC CAG GCC CAT TG

## Data Availability

Data sharing is not applicable.
